# C-Myc Expression in Oral Squamous Cell Carcinoma: Molecular Mechanisms in Cell Survival and Cancer Progression

**DOI:** 10.3390/ph15070890

**Published:** 2022-07-19

**Authors:** Guya Diletta Marconi, Ylenia Della Rocca, Luigia Fonticoli, Francesco Melfi, Thangavelu Soundara Rajan, Simone Carradori, Jacopo Pizzicannella, Oriana Trubiani, Francesca Diomede

**Affiliations:** 1 Department of Medical, Oral and Biotechnological Sciences, University “G. d’Annunzio” Chieti-Pescara, Via dei Vestini, 31, 66100 Chieti, Italy; guya.marconi@unich.it; 2 Department of Innovative Technologies in Medicine & Dentistry, University “G. d’Annunzio” Chieti-Pescara, Via dei Vestini, 31, 66100 Chieti, Italy; ylenia.dellarocca@unich.it (Y.D.R.); luigia.fonticoli@unich.it (L.F.); oriana.trubiani@unich.it (O.T.); francesca.diomede@unich.it (F.D.); 3 Department of Pharmacy, University “G. d’Annunzio” Chieti-Pescara, Via dei Vestini 31, 66100 Chieti, Italy; francesco.melfi@unich.it (F.M.); simone.carradori@unich.it (S.C.); 4 Department of Biotechnology, Karpagam Academy of Higher Education, Coimbatore 641021, India; drsoundararajan.t@kahedu.edu.in; 5 Karpagam Cancer Research Centre, Karpagam Academy of Higher Education, Coimbatore 641021, India; 6 Ss. Annunziata Hospital, ASL 02 Lanciano-Vasto-Chieti, 66100 Chieti, Italy

**Keywords:** squamous cell carcinoma, doxorubicin, tumor microenvironment, c-Myc, Cal-27

## Abstract

Oral squamous cell carcinoma (OSCC) represents 90% of malignant epithelial cancer that occurs in the oral cavity. The c-Myc factor is expressed in multiple types of cancer, comprising head and neck squamous cell carcinoma (HNSCC), where it plays a fundamental role in tumor prognosis and in the self-renewal of tumor stem cells. However, the role of c-Myc in controlling OSCC cells is not well-known. The aim of the present study is the evaluation of the biological roles and regulatory mechanism of c-Myc in the pathogenesis of OSCC. Results indicated that c-Myc, c-Jun, Bcl-2, hypoxia inducible factor-1α (HIF-1α), vascular endothelial growth factor (VEGF), matrix metalloproteinase-9 (MMP-9), ERK 1/2 and pERK1/2 were overexpressed in a cellular model of squamous cell carcinoma, Cal-27. Doxorubicin (Doxo), a common chemotherapeutic agent, inhibited cell invasion, hypoxia, angiogenesis and inflammation in a cellular model of Cal-27 cells as indicated by downregulation of MMP-9, VEGF, ERK 1/2 and pERK 1/2 as well as promoted apoptosis as evidenced by the downregulation of Bcl-2 protein. This work aimed at underlying the functional relevance of c-Myc in OSCC and the HIF-Myc collaboration by integrating the knowledge on this molecular link in an OSCC tumor microenvironment. The results obtained showed for the first time the vital role of c-Myc in Cal-27 in cell survival/proliferation and tumor growth as well as the negative regulatory effect of Doxo against c-Myc signaling pathway.

## 1. Introduction

Head and neck squamous cell carcinomas (HNSCCs) have a great incidence worldwide, with a mortality rate of 40–50%. The tumors initiate in the epithelial cells of the mucosal layers in the oral cavity, oropharynx, larynx or hypopharynx [1] and they represent the most widespread malignancies that originate in the head and neck. Head and neck carcinoma is notably heterogeneous and is characterized by different etiologies and molecular alterations [2]. Different therapeutic approaches such as chemotherapy, radiotherapy and surgery are used to treat this complex malignancy. Due to the lack of efficient therapeutic approaches, the necessity to find a valuable target molecule against HNSCCs is strongly needed. c-Fos upregulation has been found in HNSCC cells and clinical data suggest that c-Fos may be associated with lymph node metastasis in oral cancer [3,4]. c-Fos is a proto-oncogene that encodes for a nuclear DNA-binding protein, which then dimerizes with the c-Jun gene product and finally forms the transcription factor 1 activating protein (AP-1). Since the c-Fos protein is a member of the AP-1 family, it is mainly associated with signal transduction and cell differentiation/proliferation [5]. Furthermore, c-Fos is associated with lymphonoidal metastatic progression and poor differentiation, mainly in coexpression with c-Jun, especially the simultaneous co-expression of c-Jun/c-Fos/p53 in oral cell squamous carcinoma (OSCC) has been identified as a prognostic factor for survival [4]. c-Myc proto-oncogene is correlated with c-Fos and c-Jun in gene regulation for cell proliferation and this protein has many unusual features that are shared by some other oncogenes such as c-Fos. Myc, a multifunctional transcription factor, regulates many genes involved in multiple biological processes such as cell growth, proliferation and apoptosis. Moreover, it promotes invasion events by activating MEK–ERK pathways [6,7,8]. It is well-known that c-Myc is expressed in many different types of cancer, including HNSCCs, in which c-Myc overexpression is responsible for poor tumor prognosis as well as the self-renewal of tumor stem cells [9,10]. Based on the literature, oncogenic c-Myc has been shown to be necessary to maintain the constitutive level of hypoxia inducible factor-1α (HIF-1α) protein in multiple myeloma (MM) cells, and thus influences VEGF secretion and angiogenic activity [11]. The present work was aimed at studying the underlying functional relevance of c-Myc in OSCC and in the HIF-Myc collaboration in Cal-27 OSCC cell line by integrating the knowledge on the molecular links in OSCC tumor microenvironment. For this reason, the current study was based on the analysis of the molecular links of c-Myc/HIF-1α gene expression as well as on the expression of other markers such as c-Jun, ERK 1/2, pERK 1/2, VEGF, MMP-9, and Bcl-2, which are involved in Cal-27 survival/proliferation and tumor progression. Moreover, the biological effect of Doxorubicin (Doxo), a well-known chemotherapeutic agent, has been evaluated in the same pathways. Our work revealed the vital role of c-Myc/HIF-1α collaboration and the potential utilization of c-Myc as a potential target for OSCC clinical treatment.

## 2. Results

### 2.1. Effects of Doxo on Cal-27 Cell Viability

**Figure 1:**
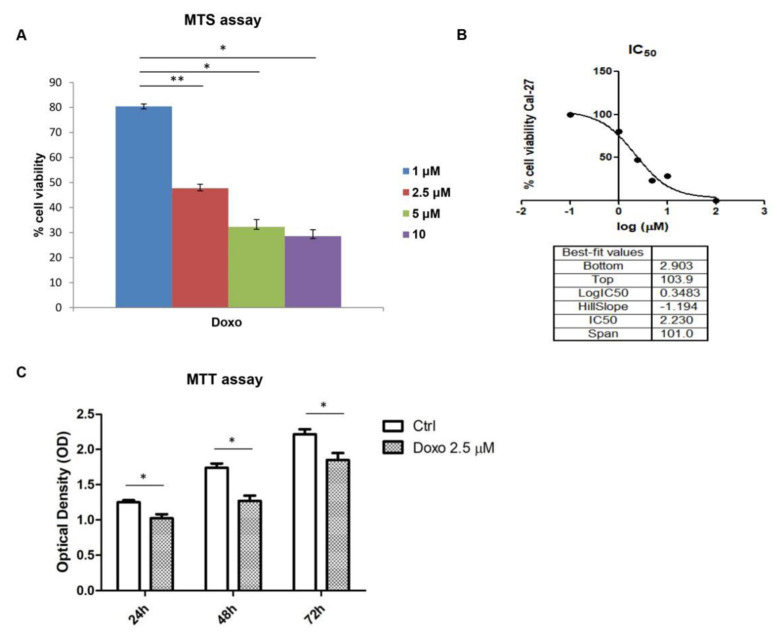
(**A**) Cell viability on Cal-27 cells treated with Doxo at 1, 2.5, 5 and 10 μM for 24 h. Cell viability was assessed using MTS assay and normalized to control cells treated with DMSO (0.2% as final concentration). ** *p* < 0.01; * *p* < 0.05. (**B**) the IC_50_ value graph for Doxo. (**C**) MTT assay reported the Doxo treatment on Cal-27 cells at 24, 48 and 72 h. * *p* < 0.05.

The effects of Doxo against the survival of Cal-27 cell line was studied by MTS assay. Initially, Doxo was tested at different concentrations ranging from 1 to 10 µM to evaluate the effect on cell viability, based on previous data obtained (data not shown). The cell-viability graph showed a cell-viability reduction in a dose-dependent manner. A total of 1 µM Doxo treatment on Cal-27 cell line showed a cell-viability percentage of 80% after 24 h, while 5 µM to 10 µM treatment exerted a cell-viability percentage of less than 50% (Figure 1A). Then, we extrapolated the IC_50_ for Doxo using Graph Pad Prism result and obtained the value of 2.5 µM as the IC_50_ value (Figure 1B). Hence, Doxo at 2.5 μM was used for further investigations. To evaluate the effects of Doxo at 2.5 μM, MTT assay was performed at 24, 48 and 72 h on Cal-27 cells. Cell viability was reduced at all considered endpoints, as reported in Figure 1C.

### 2.2. Expression Level of c-Myc, c-Jun, Bcl-2, HIF-1α, VEGF, MMP-9, ERK 1/2 and pERK 1/2 in Doxo-Treated Cells

**Figure 2:**
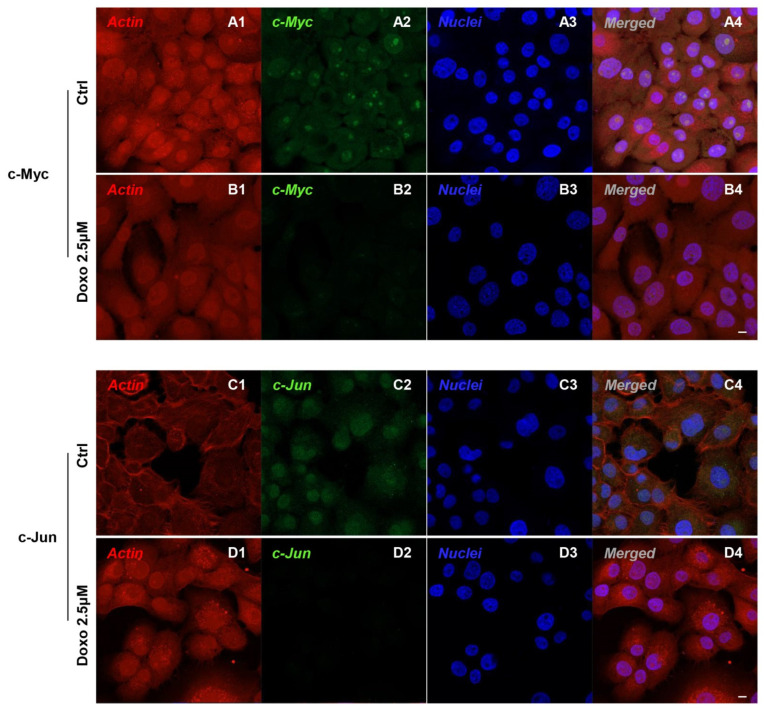
Confocal microscopic analysis on the expression of c-Myc and c-Jun in Cal-27 cell lines. Expression of c-Myc and c-Jun analyzed by confocal microscopy in untreated cells (CTRL) (**A1**–**A4**,**C1**–**C4**); expression of c-Myc and c-Jun expression in Cal-27 treated with Doxo 2.5 μM (**B1**–**B4**,**D1**–**D4**). Red fluorescence: cytoskeleton actin. Green fluorescence: c-Myc and c-Jun. Blue fluorescence: cell nuclei. Scale bar: 20 µm.

**Figure 3:**
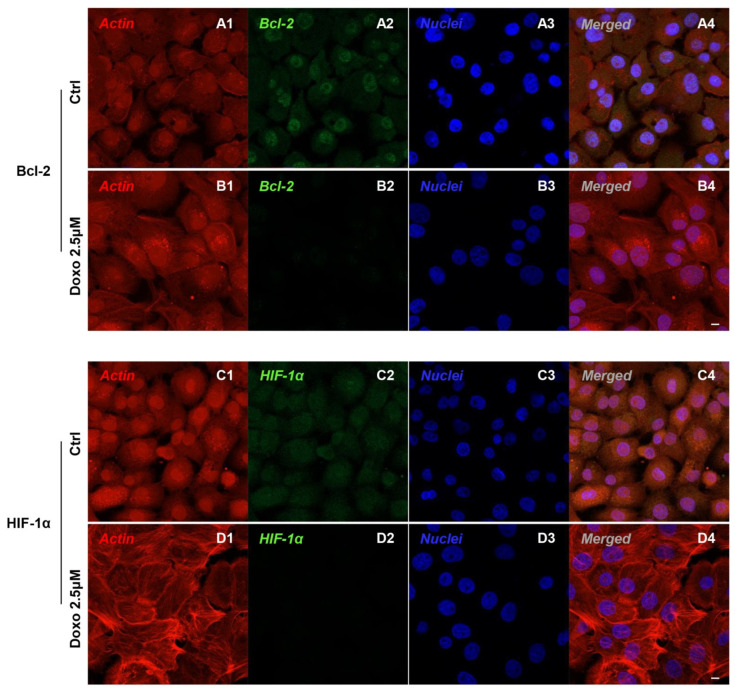
Confocal microscopic analysis on the expression of Bcl-2 and HIF-1α in Cal-27 cell lines. Expression of Bcl-2 and HIF-1α analyzed by confocal microscopy in untreated cells (CTRL) (**A1**–**A4**,**C1**–**C4**); expression of Bcl-2 and HIF-1α expression in Cal-27 treated with Doxo 2.5 μM (**B1**–**B4**,**D1**–**D4**). Red fluorescence: cytoskeleton actin. Green fluorescence: Bcl-2 and HIF-1α. Blue fluorescence: cell nuclei. Scale bar: 20 µm.

**Figure 4:**
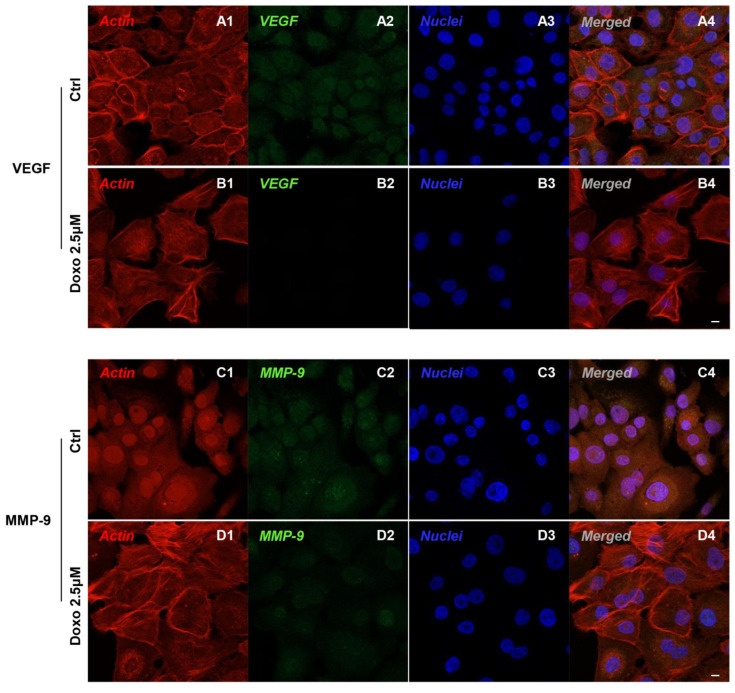
Confocal microscopic analysis on the expression of VEGF and MMP-9 in Cal-27 cell lines. Expression of VEGF and MMP-9 analyzed by confocal microscopy in untreated cells (CTRL) (**A1**–**A4**,**C1**–**C4**); expression of VEGF and MMP-9 analyzed by confocal microscopy in Cal-27 treated with Doxo 2.5 μM (**B1**–**B4**,**D1**–**D4**). Red fluorescence: cytoskeleton actin. Green fluorescence: VEGF, MMP-9, ERK 1/2 and pERK 1/2. Blue fluorescence: cell nuclei. Scale bar: 20 µm.

**Figure 5:**
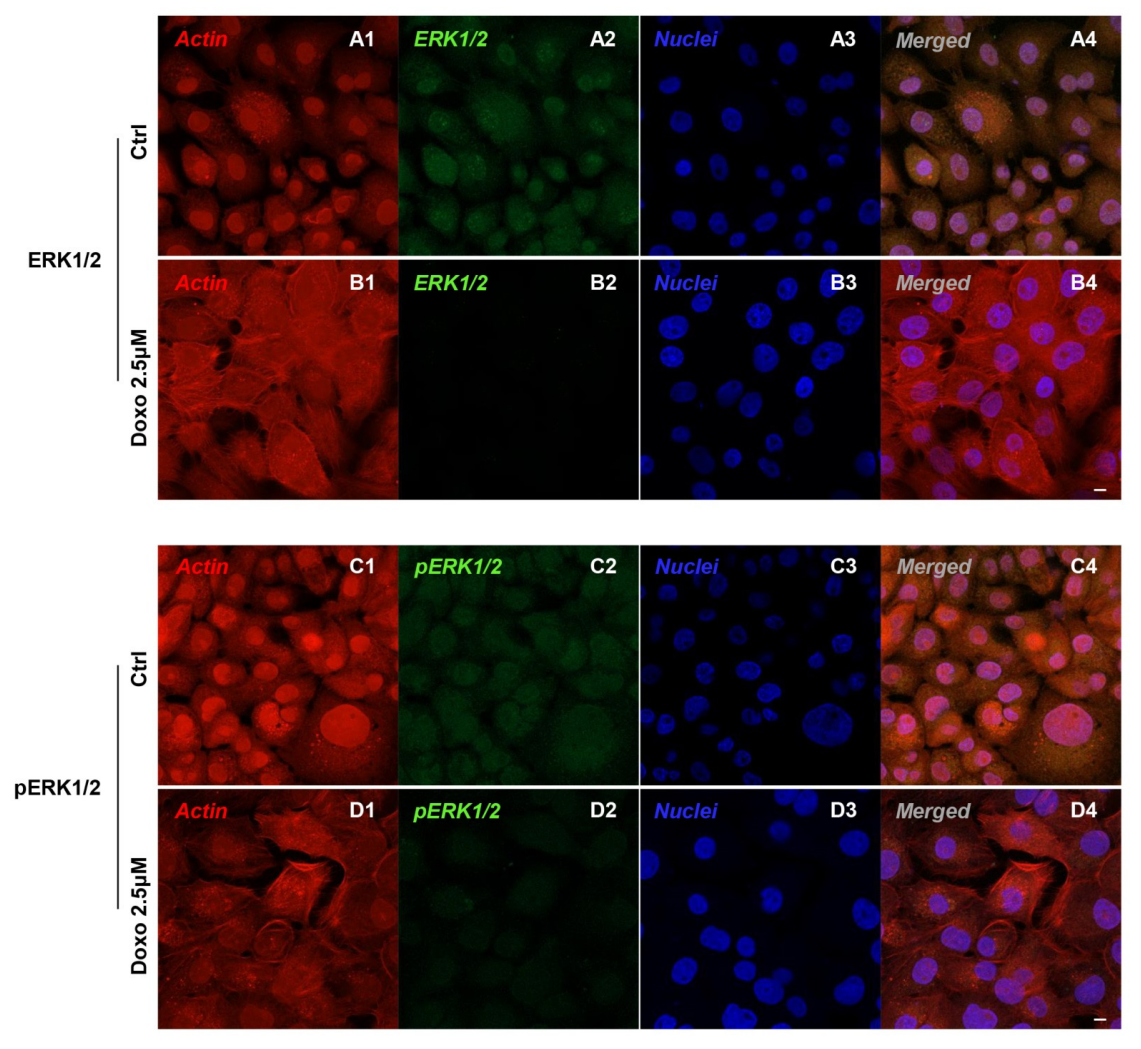
Confocal microscopic analysis on the expression of ERK 1/2 and pERK 1/2 in Cal-27cell lines. Expression of ERK 1/2 and pERK 1/2 analyzed by confocal microscopy in untreated cells (CTRL) (**A1**–**A4**,**C1**–**C4**); expression of ERK 1/2 and pERK 1/2 analyzed by confocal microscopy in Cal-27 treated with Doxo 2.5 μM (**B1**–**B4**,**D1**–**D4**). Red fluorescence: cytoskeleton actin. Green fluorescence: ERK 1/2 and pERK 1/2. Blue fluorescence: cell nuclei. Scale bar: 20 µm.

**Figure 6:**
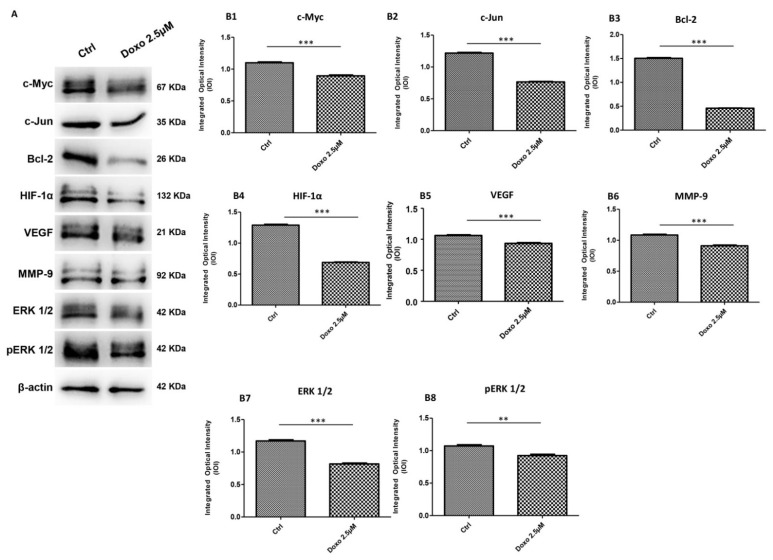
Western blotting analysis. (**A**) c-Myc, c-Jun, Bcl-2, HIF-1α, VEGF, MMP-9, ERK 1/2 and pERK1/2 proteins expression in Cal-27 cell line untreated and treated with 2.5 μM Doxo. Each membrane was probed with β-actin antibody to verify loading consistency. Western blot data shown are the representative data from three different experiments. (**B1**–**B8**) Histograms represent densitometric measurements of protein bands expressed as integrated optical intensity (IOI) mean of three separate experiments. The error bars show standard deviation (±SD). Densitometric values analyzed by *t*-test (unpaired *t*-test) return significant differences. *** *p* < 0.001, ** *p* < 0.01.

**Figure 7:**
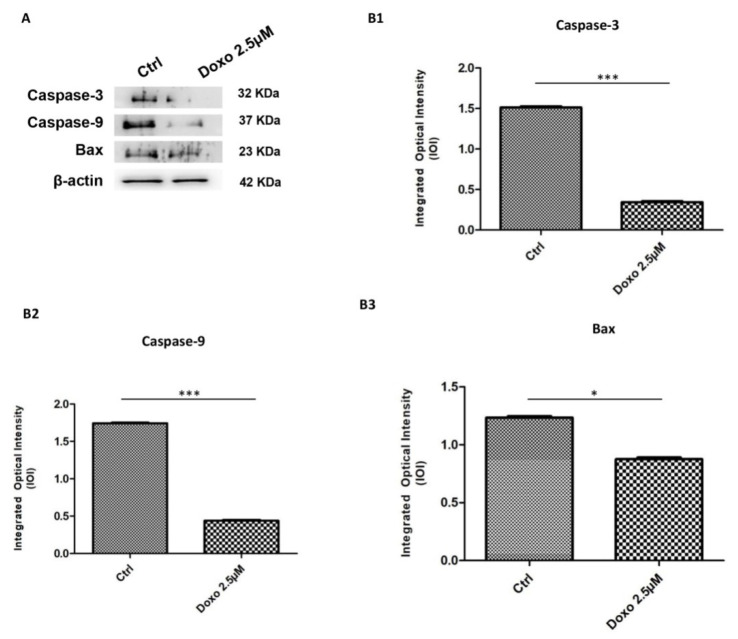
Western blotting analysis of apoptosis related markers. (**A**) Caspase-3, caspase-9 and Bax proteins expression in Cal-27 cell line untreated and treated with 2.5 μM Doxo. Each membrane was probed with β-actin antibody to verify loading consistency. Western blot data shown are the representative data from three different experiments. (**B1**–**B3**) Histograms represent densitometric measurements of proteins bands expressed as integrated optical intensity (IOI) mean of three separate experiments. The error bars show standard deviation (± SD). Densitometric values analyzed by *t*-test (unpaired *t*-test) return significant differences. *** *p* < 0.001, * *p* < 0.05.

Immunofluorescence results evidenced that c-Myc, c-Jun, Bcl-2, HIF-1α, VEGF, MMP-9, ERK 1/2 and pERK1/2 proteins were expressed significantly in Cal-27 untreated cells. On the contrary, Cal-27 cancer cells treated with 2.5 µM Doxo showed a marked reduction of these proteins compared to the untreated cells (Figure 2, Figure 3, Figure 4 and Figure 5). These results were confirmed by Western blot analysis, in which the expression of c-Myc, c-Jun, Bcl-2, HIF-1α, VEGF, MMP-9, ERK 1/2 and pERK 1/2 were significantly reduced in Doxo-treated cells compared to the untreated cells (Figure 6). To evaluate the Doxo treatment on apoptosis-related markers, Western blotting analysis was performed on Cal-27 cells to evaluate the expression of caspase-3, caspase-9 and Bax. Caspase-3, caspase-9 and Bax showed a down-expression in Cal-27 Doxo-treated cells when compared to the CTRL group, as demonstrated by protein-specific bands (Figure 7A) and these results were confirmed by densitometric analyses (Figure 7B1–B3).

## 3. Discussion

Tumor is a heterogeneous illness with complex molecular changes that consist of genetic and epigenetic alterations. OSCC is the eighth-most common oral malignancy, constituting 90% of all oral malignancies [12]. The main aspects of tumor biology are proliferation, apoptosis and differentiation. 

Although Doxo is not considered the gold standard for chemotherapy in OSCC, some studies have been reported the use of Doxo for noninvasive diagnostic imaging and therapeutical application using multifunctional nanotechnology to increase the intracellular uptake [13,14]. In tumor advancement, proto-oncogenes such as c-Myc and Bcl-2 are considered as genes that regulate cellular proliferation and apoptosis [15].

*C-MYC* gene is involved in the initiation of cyclin-dependent kinases which promote transcription followed by cellular proliferation.

Chen et al. reported that the c-Myc inhibitor 10058-F4 could work synergistically with Doxo treatment to inhibit the proliferation of drug-resistant cells and promote their apoptosis in triple-negative breast cancer (TNBC) [16]. Doxo treatment is also related to the epithelial-to-mesenchymal transition (EMT) repression through targeting c-Myc in in vitro study in lung cancer [17]. EMT is a biological process through which epithelial cells are transformed into mesenchymal-phenotype and plays a pivotal role in regulating tumor pathogenesis [18].

Gonzalez-Gonzalez et al. reported the molecular mechanism that targets the EMT process in the HNSCC [19]. During EMT, the alteration of epithelium-specific adhesion proteins and the induction of mesenchymal proteins such as matrix metalloproteinases (MMPs) was occurred. In tumor progression, the extracellular matrix (ECM) destruction and hypoxia are the main studied processes. In HNSCC MMP-9 plays an important role during tumor invasion [20], as well as the participation of ERK-1/2 and PI3K signaling pathways [21]. Several genes were reported in the promotion of carcinogenesis of HNSCC as c-Myc and cyclin D1 [22]. On the other hand, in OSCC, tumor progression and metastasis are also sustained by the presence of hypoxia-inducible factor (HIF) and related genes induced by hypoxia, such as VEGF, interleukin 1A (IL-1A), endothelin 1, platelet-derived growth factor B (PDGFB) and erythropoietin (EPO) [23].

*BCL2* gene was first discovered in follicular B-cell lymphoma, which is connected to the immunoglobulin heavy-chain locus at the breakpoints of t(14;18) translocation. The effect of this translocation is the induction of Bcl-2 protein transcription. Bcl-2 protein preserves membrane integrity of mitochondria and was found to inhibit cell death [24]. *C-MYC* and *BCL2* oncogenes evidence parallel outcomes in cancer development. Previous studies documented the synergistic mechanism of Bcl-2 and c-Myc in the abrogation of apoptosis and facilitating abnormal proliferation of the cell. The role of these genes is still unspecified in the progression, aggressiveness and prognosis in OSCC. The role of *C-MYC* in regulating the proliferation of OSCC cells is not well-identified [25]. The antiapoptotic Bcl-2 protein is overexpressed in cancer cells, as demonstrated in the Intrahepatic cholangiocarcinoma (iCCA) model, in which the increase expression of BCL-2 and c-FLIP is associated with the reduction in apoptosis due to the lack of activation of the caspase cascade [26]. Our results showed an overexpression of Bcl-2, caspase-3, caspase-9 and Bax in untreated Cal-27 cells, while Doxo treatment showed a reduction in all considered proteins.

Emerging studies suggest that c-Myc and HIF also collaborate to induce cancer cell growth and progression [27]. C-Myc signaling plays a key part in controlling cancer-cell metabolism and vasculogenesis. The Myc oncoprotein is a master regulator of transcription that activates or represses gene expression to coordinate diverse cellular processes, including cellular differentiation, apoptosis and angiogenesis [28]. C-Myc and HIFs also cooperate to promote tumor angiogenesis and metastasis. Under hypoxic conditions, HIF-1α can stimulate the expression of various proangiogenic factors, including VEGF and matrix metalloproteinases (MMP-2 and MMP-9). Based on the literature, oncogenic c-Myc cooperates with HIF-1α to trigger VEGF production and release [29,30].

Our results showed the down-expression of c-Myc and HIF-1α after Doxo treatment in Cal-27 cells. It is also possible that the Myc-HIF link further interplays with other oncogenic pathways such as ERK/MAPK, Akt/mTOR and Notch signaling to alter cell metabolism, cell cycle, ribosome biogenesis and genomic stability in tumorigenesis. 

High levels of c-Jun are found in multiple types of cancer [31], and c-Jun overexpression might lead to an overexpression of c-Myc, since in other types of tumors it has been shown that c-Jun controls the exposure of c-Myc binding directly to its promoter, and that induces an overexpression of c-Jun accelerating the c-Myc promoter activity [32]. Based on this knowledge, the c-Myc expression in a cellular model of squamous cell carcinoma, Cal-27, was investigated. The activity of the Raf/MEK/ERK signaling pathway plays an important role in the processes of proliferation, survival and metastasis in several types of cancers, in which ERK 1/2 are activated through phosphorylation by MAPK/ERK kinases, which are activated through phosphorylation by the upstream serine/threonine Raf protein kinases [33]. The KRAS mutant activates the Raf/MEK/ERK signaling pathway to upregulate c-Myc. In many types of cancer it has been shown that ERK is involved in the increase in c-Myc expression associated with development, growth and invasion [34]. Furthermore, the EGF receptor also activates the PI3K pathway, which is always correlated with c-Myc to stimulate the proliferation of tumor cells [35]. 

In the present study, we focused our attention on the biological roles and regulatory mechanism of c-Myc in OSCC. Obtained data showed that that c-Myc, c-Jun, Bcl-2, HIF-1α, VEGF, MMP-9, ERK 1/2 and pERK 1/2 were overexpressed in a cellular model of OSCC, Cal-27, as reported by confocal microscopy observations and protein-expression analysis.

All these proteins are associated with cell invasion, hypoxia, angiogenesis, migration and inflammation. On the other hand, Cal-27 cells treated with Doxo, at a concentration of 2.5 μM (IC_50_ value obtained), showed a down-expression of the above-mentioned proteins.

The current study explores one of the molecular mechanisms targeting oral squamous cell carcinoma cells. Novel ideas are provided by an in-depth exploration of its signal pathways for treating OSCC from the molecular level. In conclusion, the functional relevance of c-Myc in OSCC and in the Myc-HIF collaboration by integrating the knowledge on this molecular link in the OSCC tumor microenvironment was investigated. The outcomes achieved showed for the first time the pivotal role of c-Myc in Cal-27 in cell survival/proliferation and cancer development as well as the inhibitory consequence of Doxo in downregulating the signaling pathways stimulated by c-Myc. These data suggest a possible route for the control of OSCC, even if further insights into the understanding of the Myc-HIF interplay are necessary for developing novel targeted therapeutics.

## 4. Materials and Methods

### 4.1. Reference Compound

Doxorubicin hydrochloride (98.0–102.0% purity as assessed by HPLC) was purchased from Sigma-Aldrich (Milan, Italy).

### 4.2. Cell-Culture Establishment

Cal-27 cells (ATCC, Manassas, Virginia, VA, USA) were maintained in Dulbecco’s Modified Eagle’s Medium (DMEM) supplemented with 10% fetal bovine serum (Euroclone, Milan, Italy). Cultures were incubated at 37 °C in a humidified atmosphere of 5% CO_2_ in air and subcultured when they were 80% confluent.

### 4.3. Experimental Study Design

All experiments were performed in triplicate. The study was designed as follows:

(i) Untreated Cal-27, used as negative control (CTRL);
(ii) Cal-27 treated with Doxo (2.5 μM) for 24 h.

### 4.4. Cell-Viability Assay

The Cal-27 cancer cells were seeded at the cell density of 15,000/well in a 96-well tissue culture plate. The cell viability was evaluated through the 3-(4,5-dimethylthiazol-2-yl)-5-(3-carboxymethoxyphenyl)-2-(4-sulfo-phenyl)-2*H*-tetrazolium (MTS) assay (CellTiter 96^®^ Aqueous One Solution Cell Proliferation Assay, Promega, Madison, WI, USA). After 24 h of treatment with Doxo at 1, 2.5, 5 and 10µM concentration, 20 μL/well of MTS dye solution was added to culture medium, and cells were incubated for 3 h at 37 °C [36]. The amount of formazan product is directly proportional to the number of living cells in culture and it was detected by absorbance measurements at 490 nm wavelength utilizing the Synergy™ HT Multi-detection microplate reader (Biotech, Winooski, VT, USA). The MTS assay was executed in three independent experiments. To evaluate the Doxo effects at 2.5 µM concentration on Cal-27 cells3-(4,5-dimethyl-2-thiazolyl)-2,5-diphenyltetrazolium bromide (MTT) assay was used with a previously reported methodology [37].

### 4.5. Confocal Microscopy (CLSM)

The Cal-27 cells were seeded at 40,000/well on 8-well culture glass slides (Corning, Glendale, AZ, USA) treated with Doxo at 2.5 μM for 24 h. Then, the samples were fixed for 1 h with 4% paraformaldehyde in 0.1 M PBS (pH 7.4) (Lonza, Basel, Switzerland) at room temperature. After washing, cell samples were processed for the immunofluorescence staining as previously described [38]. Successively, the cells were permeabilized with 0.5% Triton X-100 in PBS (Lonza) for 10 min and blocked with 5% skimmed milk in PBS for 1 h. Then, the cells were incubated with the primary antibodies for 2 h at room temperature. The following primary antibodies were used in this experiment: anti-c-Myc (1:200) (sc-293072, Santa Cruz Biotechnology, Dallas, TX, USA), anti-c-Jun (1:200) (sc-8008, Santa Cruz Biotechnology), anti-Bcl-2 (1:200) (sc-134306, Santa Cruz Biotechnology), anti-HIF-1α (1:200) (sc-53546, Santa Cruz Biotechnology), anti-VEGF (1:200) (sc-57496, Santa Cruz Biotechnology), anti-MMP-9 (1:200) (sc-21733, Santa Cruz Biotechnology), anti-ERK 1/2 (1:200) (sc 514302, Santa Cruz Biotechnology) and anti-pERK 1/2 (1:200) (sc-81492, Santa Cruz Biotechnology). Next, samples were incubated with Alexa Fluor 488 green fluorescent conjugated goat antirabbit secondary antibody (1:200) (A32731, Molecular Probes, Invitrogen, Eugene, OR, USA) or Alexa Fluor 488 green fluorescent conjugated goat antimouse secondary antibody (1: 200) (A32723, Invitrogen, Eugene, OR, USA) for 1 h at 37 °C. To stain the cytoskeleton actin, cells were treated with Alexa Fluor 568 phalloidin red conjugate (1:200) (A12380, Invitrogen, Eugene, OR, USA) for 1 h, and to stain the nuclei, cells were stained with TOPRO (1:200) (T3605, Invitrogen, Eugene, OR, USA) for 1 h. The Zeiss LSM800 confocal system (Carl Zeiss, Jena, Germany) was used to acquire microphotographs.

### 4.6. Western Blot

The cell lysates (50 µg) underwent electrophoresis and transferred to the polyvinylidenfluoride (PVDF) membrane. Successively, they were blocked in 5% of non-fat milk in PBS + 0.1% Tween-20. Then, the blotted membranes were incubated overnight at 4 °C with primary antibodies of anti-c-Myc (1:500) (sc-40, Santa Cruz Biotechnology, Dallas, TX, USA), anti-c-Jun (1:500) (397500, Life Technologies), anti-Bcl-2 (1:500) (sc-7382, Santa Cruz Biotechnology), anti-HIF-1α (1:500) (sc-53546, Santa Cruz Biotechnology), anti-VEGF (1:500) (sc-57496, Santa Cruz Biotechnology), anti-MMP-9 (1:500) (sc-21733, Santa Cruz Biotechnology), anti-ERK 1/2 (1:500) (sc-514302, Santa Cruz Biotechnology), anti-pERK1/2 (1:500) (sc-81492, Santa Cruz Biotechnology), anti-caspase-3 (1:500) (sc-56052, Santa Cruz Biotechnology), anti-caspase-9 (1:500) (sc-56076, Santa Cruz Biotechnology) and anti-Bax (1:500) (sc-493, Santa Cruz Biotechnology). β-actin was used as a loading control (1:750, Santa Cruz Biotechnology). After five washings in PBS containing 0.1% Tween-20, membranes were incubated for 1 h at room temperature with peroxidase-conjugated antimouse secondary antibody (A90-116P Goat antimouse; 1:5000 dilution in 2.5% milk made by 1X PBS and 0.1% Tween-20). Protein expression was visualized using the enhanced chemiluminescence detection method (ECL) (Amersham Pharmacia Biotech, Milan, Italy) with photo documenter Alliance 2.7 (Uvitec, Cambridge, UK). Signals were analyzed by ECL enhancing and evaluated through UVIband-1D gel analysis (Uvitec, Cambridge, UK). Data were normalized with densitometric values derived from β-actin, the loading control.

### 4.7. Statistical Analysis

Statistical significance was analyzed with GraphPad Prism 5 (GraphPad, San Diego, CA, USA) software using *t*-test. Values of *p* < 0.05 were considered statistically significant.

## 5. Conclusions

The present work reported the regulatory role of c-Myc in cell survival/proliferation and cancer progression in Cal-27 cells. Moreover, our results showed that Doxo treatment downregulated the activation of the c-Myc signaling pathway.

## Data Availability

Data is contained within the article.
